# Retrospective review of patient factors impacting free-breathing respiratory motion magnitude for MR-guided radiotherapy

**DOI:** 10.1016/j.tipsro.2025.100339

**Published:** 2025-08-28

**Authors:** Mairead Daly, Holly Egan, Ananya Choudhury, Ganesh Radhakrishna, Cynthia L. Eccles

**Affiliations:** a Faculty of Biology, Medicine and Health, The University of Manchester, UK; b The Christie NHS Foundation Trust, Manchester, UK

**Keywords:** Radiotherapy, SABR, Motion, MR-guided radiotherapy, MR Linac, Abdominal cancer

## Abstract

Patient factors may influence motion management strategy selection for abdominal stereotactic ablative radiotherapy (SABR). This retrospective evaluation investigated the impact of body mass index (BMI), sex, and cirrhosis in 16 patients imaged with cine-magnetic resonance imaging (MRI) on a 1.5 T magnetic resonance linear accelerator (MR Linac). Male patients, those with cirrhosis, and higher BMI tended to exhibit greater motion magnitude, although this was not statistically significant, suggesting that these patients should be prioritised for motion management strategies. Further investigation in larger cohorts is warranted to confirm these trends and guide personalised motion management in abdominal radiotherapy.

## Highlights

• Higher BMI was linked with greater free-breathing motion magnitude.
• Patients with cirrhosis had larger respiratory motion than those without.
• Male patients showed greater motion variation than females.
• Motion management is advised for high-BMI, cirrhotic, or male patients.

## Introduction

Pancreatic cancer and hepatocellular carcinoma (HCC) account for less than 7.5 % of global cancer cases and have poor outcomes despite advances in treatment [1]. Incidence of both is typically higher in males [2,3]. Accurate patient positioning is crucial in abdominal stereotactic ablative radiotherapy (SABR), as respiratory motion can reduce dose to targets, increase dose to organs at risk (OARs), and reduce image quality [4]. Limited research exists on which patient-related factors influence respiratory motion.

Common respiratory motion management strategies include abdominal compression (AC), and breath hold (BH), and free-breathing with an internal target volume (ITV). Institutions typically adopt one or two strategies based on technical requirements, cost, and ease of implementation [5]. AC devices, including pneumatic belts, restrict abdominal motion, and for BH, treatment is initiated when the patient holds their breath. However, not all motion management strategies are universally suitable, as AC can increase motion in rare cases [6], and some patients may struggle with BH due to the repeated breath holds required per session [7].

Certain patient-related factors may impact respiratory mechanics. For example, obesity may affect breathing patterns by exerting increased pressure on the diaphragm and ribcage [8], though previous work found that subcutaneous and visceral fat − associated with obesity − did not reduce AC effectiveness [9]. Sex-based differences in respiratory kinematics, such as lung size, ribcage anatomy, and tidal volume, may also affect motion magnitude and variability [10,11]. For instance, males typically have a larger diaphragm surface area and greater ribcage expansion during breathing [[12], [13], [14]]. Cirrhosis, which is common in HCC, increases liver stiffness which can lead to greater liver motion [15]. However, the impact of obesity, sex, and cirrhosis on free-breathing motion is unknown.

This study assessed whether BMI, sex, and cirrhosis affect free-breathing respiratory motion for abdominal radiotherapy using cine-magnetic resonance imaging (MRI).

## Materials and methods

### Patients and imaging

This retrospective evaluation included sixteen abdominal radiotherapy who underwent MRI under two institutional ethics board-approved imaging studies. Six patients were imaged on PRIMER (NCT02973828 [16]), ten on QUANTUM (NCT04748094 [17]), and all were recruited chronologically with informed consent. As this is the first study of its kind, no statistical power calculation was performed. The sample size was kept intentionally small based on available imaging to determine whether further, larger-scale investigations are warranted. Each patient underwent 1–3 cine imaging sessions, with only one session per patient being included in this work. Imaging was conducted as detailed previously [9]. In brief, a single-slice balanced steady-state free-procession gradient echo (bFFE) cine-MRI was acquired in the treatment position (supine, and arms up or down based on comfort) in free-breathing on a 1.5 T Unity MR Linac (Elekta, Crawley, UK). Imaging parameters were: echo time (TE) = 1.34 ms, repetition time (TR) = 2.7 ms, flip angle = 40°, field of view (FOV) = 10 × 448 × 400 mm, matrix 132 × 150, pixel size 3 × 3 × 10 mm. The first six patients were imaged only in the coronal plane, with a total of 250 dynamic images acquired over an acquisition time (TA) of 1 m 46 s per plane at approximately 2.36 frames per second (FPS). The subsequent 10 were imaged using a 3-plane cine, acquired over 5 m 18 s with the same temporal resolution. For this study we used the coronal images only, placed intersecting the liver hilum, as they were common for all patients and showed both craniocaudal and mediolateral motion.

### Patient factors

Height, weight, self-reported sex, treatment site and cirrhosis status was retrospectively collected from the patients’ electronic health records. As patients were referred chronologically and sample sizes kept deliberately small, equal numbers of male and female patients could not be included. Cirrhosis was documented as a binary variable (yes/no), for patients with HCC this was documented as ‘yes’ if missing in the records, as it is commonly present at diagnosis [18]. BMI was calculated using the formula: weight (kg) ÷ [height (m)]^2^ [19], and categories assigned according to World Health Organization (WHO) guidelines defined in section 2.4.

### Motion quantification

**Fig. 1:**
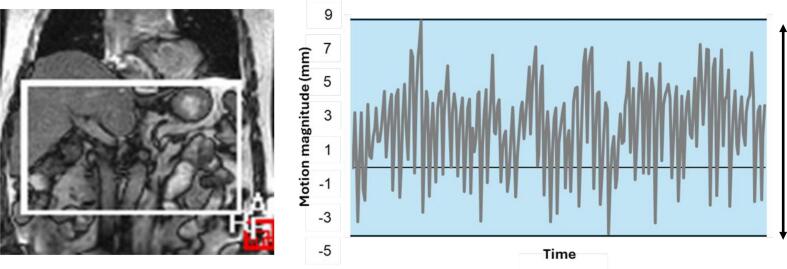
Left, sample registration clipbox on a single coronal cine-MRI for a patient in the registration software MatPel. Right, sample of raw motion data plotted as pseudo-motion trace, the arrows and shaded area represent the maximum range of motion used in this study.

Motion was quantified in the craniocaudal and mediolateral directions on one coronal cine image dataset per patient using software developed at the Netherlands Cancer Institute (MatPel, V3.9, NKI, Amsterdam, Netherlands). Motion magnitude was quantified by automatic rigid registration of the initial cine-MRI with subsequent images by a user-defined clipbox. The clipbox was set by a single user (MD) to include all visible central upper abdominal anatomy including the portal vein, inferior liver border, and stomach. Clipbox size and placement were selected on a patient-specific basis according to visible anatomy, an example is presented in Fig. 1. Registrations were visually inspected and qualitatively evaluated by a single trained observer (MD) for consistency. Intra-observer registration variation was not assessed. Registration displacements were plotted in Excel, generating a pseudo-motion trace (Fig. 1), which was visually assessed and major outliers excluded (i.e., sneeze, registration error). The range between maximum and minimum displacements from this pseudo-motion trace data was calculated to provide the maximum respiratory amplitude.

### Analysis

Variables including age, motion magnitude and BMI were summarised using descriptive statistics. We reported median, range, and IQR to capture both data spread and outliers, which was important given the small sample size. To analyse the impact of BMI on motion magnitude and variability, patients were assessed as a single group and subdivided based on BMI according to the WHO classification system: ‘underweight’ (BMI < 18.5 kg/m^2^), ‘normal’ (BMI 18.5–24.9 kg/m^2^), ‘overweight’ (BMI 25–29.9 kg/m^2^), or ‘obese’ (BMI > 30 kg/m^2^) [19]. A Mann-Whitney *U* test was used to assess differences in sex (male/female) and cirrhosis (yes/no) subgroups. A p-value of less than 0.05 was considered statistically significant for a 5 % probability of Type 1 error. Differences in motion magnitude between the three BMI categories were assessed using a Kruskal-Wallis test. To determine the influence of each patient factor evaluated (e.g., BMI) on magnitude and variability of respiratory motion, a two-tailed Spearman Rank Correlation Test was performed. Due to the small sample size and uneven subgroup distribution, multivariable analysis was not performed.

## Results

**Fig. 2:**
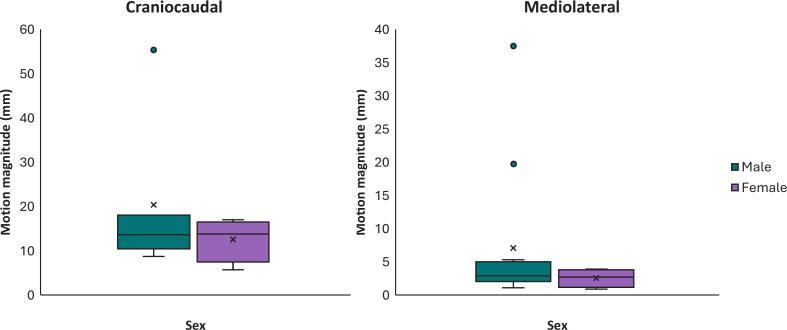
Boxplot of craniocaudal (left) and mediolateral (right) motion magnitude in millimetres (mm) measured on cine-MRI for both sexes. There was no statistical difference found using Mann-Whitney *U* test between both groups for both craniocaudal (*p* = 0.95) or mediolateral (*p* = 0.36) motion. Note: the major ‘male’ outlier in both figures corresponds to the same patient.

Of the sixteen patients included in this work, seven received radiotherapy to the liver, six to the pancreas, two to abdominal node oligometastases, and one to an adrenal gland metastasis. Patient characteristics are summarised in Table 1. Overall median (range, interquartile range − IQR) free-breathing craniocaudal respiratory motion magnitude was 13.1 mm (range 5.7–55.5 mm, IQR 10.6–16.4 mm). The two extreme outliers, with craniocaudal motion of 55.4 and 55.5 mm, were both male. The median craniocaudal motion magnitude was larger for the four females at 13.8 mm (range 5.7–17.0 mm, IQR 10.9–15.4 mm, Fig. 2) than for the twelve males at 12.2 mm (range 8.7–55.5 mm, IQR 10.6–16.6 mm), although there was a larger range of motion for males, and the group sizes were not matched. Differences between motion magnitude across sexes were not statistically significant on Mann-Whitney *U* test (p = 0.95). Median mediolateral motion magnitude was similar between males (2.9 mm, range 1.1–37.5 mm, IQR 2.1–4.4 mm) and females (2.7 mm, range 0.9–3.9 mm, IQR 1.7–3.6 mm; p = 0.36), although males again exhibited a wider range.

**Table 1:** Characteristics of included patients. BMI = body mass index, mm = millimetres, n = number, SD = standard deviation, HCC = hepatocellular carcinoma.

		N, median	%, range
Participants (n, %)	Total	16	100.0
Sex *(n, %)*	Male	12	75.0
	Female	4	25.0
Age, years *(median, range)*	Years	63.5	41–82
BMI *(n, %)*	18.5–24.9	6	37.5
	25–29.9	6	37.5
	>30	4	25.0
Treatment site *(n, %)*	Liver	7	43.8
	*HCC*	*3*	*42.9*
	*Metastasis*	*4*	*57.1*
	Pancreas	6	37.5
	Abdominal node	2	12.5
	Adrenal gland	1	6.3
Motion magnitude, mm *(median, range)*	Craniocaudal	13.1	5.71–55.5
	Mediolateral	2.9	0.9–37.5

**Fig. 3:**
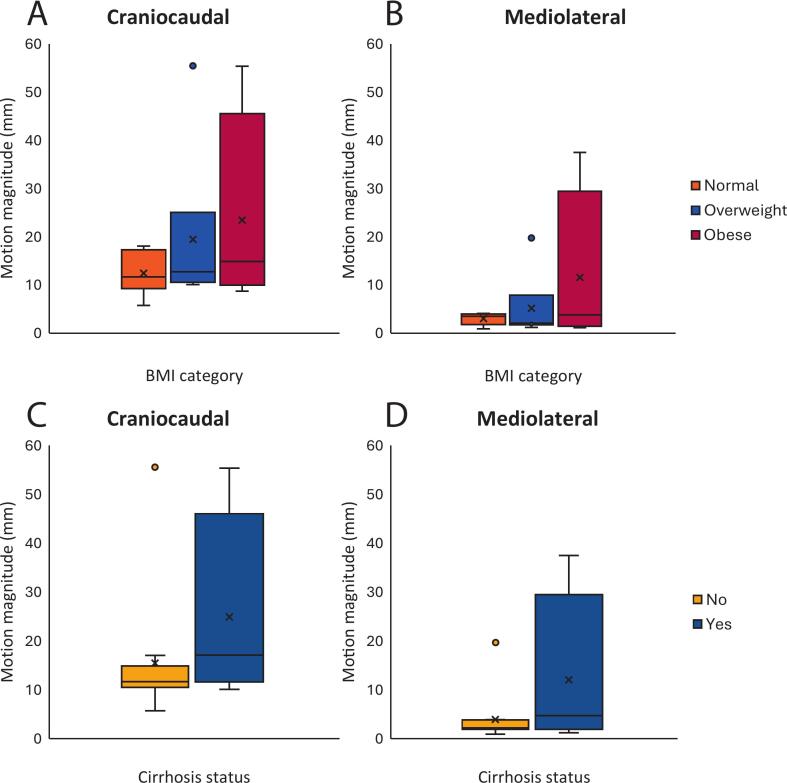
Boxplot of craniocaudal (A) and mediolateral (B) motion magnitude across BMI groups, and craniocaudal (C) and mediolateral (D) motion magnitude for patients with and without cirrhosis. There was no statistical difference found between BMI groups (A & B) for both craniocaudal (p = 0.85) and mediolateral (p = 0.74) motion, and none found between cirrhosis groups for both craniocaudal (p = 0.26) or mediolateral (p = 0.16) motion. Note: two extreme outliers Note: two extreme outliers were identified across the groups (excluding ‘normal’ BMI), corresponding to the same patients in Figures A and B, and Figures C and D. One outlier appears as a point beyond the upper whisker; the other, while within the whisker range, still represents an extreme value.

Median craniocaudal motion magnitude was 11.7 mm (range 5.7–18.1 mm, IQR 10.5–15.9 mm) for ‘normal’ BMI, 12.7 mm (range 10.1–55.5 mm, IQR 10.7–14.9 mm) for ‘overweight’, and 14.9 mm (range 8.7–55.4 mm, IQR 12.4–25.9 mm) for ‘obese’ categories (Fig. 3A and B). The maximum free breathing motion in the mediolateral direction was larger in patients classified as ‘overweight’ and ‘obese’ than ‘normal’ BMI, with median motion of 2.1 mm (range 1.2–19.7 mm, IQR 1.9–3.5 mm), 3.8 mm (range 1.1–37.5 mm, IQR 2.0–13.4 mm), and 3.5 mm (range 0.9–4.1 mm, IQR 2.5–3.8 mm), respectively. Although motion magnitude appeared to increase with BMI, no statistically significant relationship was found using a Kruskal-Wallis test for either craniocaudal (p = 0.85) and mediolateral (p = 0.74) motion across the three groups. Additionally, no relationship was seen using two-tailed Spearman Rank Correlation Test between BMI and either craniocaudal (r = 0.11) or mediolateral (r = 0.08) motion.

Respiratory motion was larger for patients with cirrhosis than without, although the group sizes for both were not matched. Median craniocaudal motion was 17.1 mm (range 10.1–55.4 mm, IQR 14.6–27.4 mm) for patients with cirrhosis, and 11.7 mm (range 5.7–55.5 mm, IQR 10.6–14.8 mm) for those without (Fig. 3C and D), although this was not statistically significant (p = 0.26); one extreme value was present in each group and is visible in the figure. This was similar for the mediolateral direction, with median motion of 4.7 mm (range 1.2–37.5 mm, IQR 3.4–13.4 mm) for patients with cirrhosis, and 2.2 mm (range 0.9–19.7 mm, IQR 2.0–3.6 mm, p = 0.16) for those without.

## Discussion

This is the first study to evaluate the impact of sex, BMI, and cirrhosis on free-breathing motion for abdominal SABR. While differences in median motion were small, greater range in motion was noted in male patients, those with cirrhosis, and patients with higher BMI. These findings, though not statistically significant, may be clinically relevant where alternatives to free-breathing with the ITV approach are not available.

Despite their potential impact on respiration, patient characteristics are often underreported in radiotherapy motion management studies [6], limiting understanding of the complex relationship between patient comfort, body composition, and respiratory mechanics. For example, obesity can alter respiratory mechanics, reducing the contribution of intercostal muscles in the supine position [20], and increasing respiratory rate [8]. One study using AC found increased respiratory motion was associated with higher BMI [21], whereas another report found no relationship [9]. Sex-based differences in respiratory mechanics – such as diaphragm size and thorax shape – are well-documented [22,23], but underexplored in radiotherapy contexts. Our findings align with existing data, with males tending to exhibit larger motion magnitudes, although not statistically significant in this sample. The present study found slightly larger, but not statistically significant, motion in patients with cirrhosis similar to a previous study [11], suggesting further investigation is warranted. Furthermore sarcopenia, associated with diaphragm atrophy and not assessed in this study, may also impact respiratory mechanics [24].

Motion magnitude and variability affects free-breathing treatment and can also reduce the efficiency of gated or tracked treatments. Our findings support further evaluation of individualised motion management strategy selection in abdominal SABR. Understanding patient-specific factors affecting respiratory motion may aid resource allocation. Regardless of strategy, respiratory motion should be monitored throughout treatment, such as with image-guided radiotherapy (IGRT), where possible.

This study’s strengths include diverse patient characteristics, representative of the typical cohort at our institution, and evaluation of motion in two anatomical planes. However, the findings of this study should be interpreted in the context of its limitations. First, the sample size was kept deliberately small, limiting the power of both overall and subgroup analysis, so any true effect may have been smaller than the study was powered to detect. Motion was analysed in the coronal plane only, therefore, not evaluating anteroposterior motion, and non-rigid motion was not assessed. The heterogeneity of included treatment sites may have introduced variability in the results. Finally, pulmonary function, which can influence respiratory motion, was not evaluated.

Future studies should:

• Increase sample size to improve power in detection of differences between cohorts.
• Incorporate multi-institutional design to improve generalisability of results.
• Record and analyse key characteristics (e.g., BMI, cirrhosis, comorbidities, anxiety, sarcopenia), along with presence of pulmonary conditions including chronic obstructive pulmonary disease (COPD), and medications (i.e., benzodiazepines).
• Consider use of spirometry to provide further insight into the interplay between lung function and motion.

## Conclusion

Patients who are male, have cirrhosis, or high BMI demonstrated greater free-breathing motion magnitude and variability on cine-MRI, although not statistically significant in this small sample. Our results suggest that these groups may benefit most from respiratory motion management in abdominal SABR. Availability of multiple strategies in departments enables personalised treatment. Larger studies are needed to confirm these findings and refine patient selection criteria for motion management in MR-guided radiotherapy.

## Informed patient consent

The author(s) confirm that written informed consent has been obtained from the involved patient(s) or if appropriate from the parent, guardian, power of attorney of the involved patient(s); and, they have given approval for this information to be published in this case report (series).

## Funding

Mairead Daly is supported by Cancer Research UK via funding to the Cancer Research UK Manchester Radiation Research Centre of Excellence [RRCOER-Jun24/100008], the NIHR Manchester Biomedical Research Centre (NIHR203308), and The Christie Hospital Charitable Fund. Ananya Choudhury and Cynthia Eccles are supported by NIHR Manchester Biomedical Research Centre (NIHR203308). This work is also supported by Cancer Research UK Manchester Centre (CTRQQR-2021\100010).

## Data sharing

Anonymized research data are stored in an institutional repository and can be shared upon formal request to the corresponding author.

## Declaration of competing interest

The authors declare that they have no known competing financial interests or personal relationships that could have appeared to influence the work reported in this paper.
